# Assessment of operators’ mental workload using physiological and subjective measures in cement, city traffic and power plant control centers

**DOI:** 10.15171/hpp.2016.17

**Published:** 2016-06-11

**Authors:** Majid Fallahi, Majid Motamedzade, Rashid Heidarimoghadam, Ali Reza Soltanian, Shinji Miyake

**Affiliations:** ^1^Department of Occupational Hygiene, Hamadan University of Medical Sciences, Hamadan, Iran; ^2^Department of Ergonomics, Research Center for Health Sciences, Hamadan University of Medical Sciences, Hamadan, Iran; ^3^Department of Ergonomics, Medical Sciences Research Center, Hamadan University of Medical Sciences, Hamadan, Iran; ^4^Modeling of Noncommunicable Diseases Research Center, Department of Biostatistics and Epidemiology, Hamadan University of Medical Sciences, Hamadan, Iran; ^5^School of Health Sciences, University of Occupational & Environmental Health, Japan, 1-1 Iseigaoka, Yahatanishiku, Kitakyushu 807-8555, Japan

**Keywords:** Mental workload, ECG, EEG, NASA-TLX

## Abstract

**Background: ** The present study aimed to evaluate the operators’ mental workload (MW) of cement, city traffic control and power plant control centers using subjective and objective measures during system vital parameters monitoring.

**Methods:** This cross-sectional study was conducted from June 2014 to February 2015 at the cement, city traffic control and power plant control centers. Electrocardiography and electroencephalography data were recorded from forty males during performing their daily working in resting, low mental workload (LMW), high mental workload (HMW) and recovery conditions (each block 5 minutes). The NASA-Task Load Index (TLX) was used to evaluate the subjective workload of the operators.

**Results:** The results showed that increasing MW had a significant effect on the operators subjective responses in two conditions ([1,53] = 216.303, P < 0.001, η2 = 0.803). Also,the Task-MW interaction effect on operators subjective responses was significant (F [3, 53] = 12.628,P < 0.001, η2 = 0.417). Analysis of repeated measures analysis of variance (ANOVA) indicated that increasing mental demands had a significant effect on heart rate, low frequency/high frequency ratio, theta and alpha band activity.

**Conclusion:** The results suggested that when operators’ mental demands especially in traffic control and power plant tasks increased, their mental fatigue and stress level increased and their mental health deteriorated. Therefore, it may be necessary to implement an ergonomic program or administrative control to manage mental probably health in these control centers. Furthermore, by evaluating MW, the control center director can organize the human resources for each MW condition to sustain the appropriate performance as well as improve system functions.

## Introduction

The concept of mental workload (MW) has become an important issue for all kinds of industry since1960s.^1^ Many ergonomists or researchers have applied subjective and physiological measures to evaluate MW quantitatively.^2-4^ Subjective rating scales, as an important tool, are used to evaluate MW of system operators.^5^ de Winter^6^ has explained that the most frequently used subjective rating scales are the NASA-Task Load Index (TLX).^7^ Psychophysiological measures allow a more objective workload assessment and can provide “real time” evaluation.^8^ Heart rate or heart rate variability (HRV) collected from an electrocardiogram (ECG) is widely used to evaluate MW^9,10^ and recording of signal is noninvasive and safe; it causes no injuries or pain to humans.^11^ Also, most studies apply information from the frequency bands of the EEG to analyze MW and fatigue.^12^ MW will lead to changes of EEG components: alpha band, beta band, theta band, and delta band.^13^ EEG signals can be acquired outside of specialized laboratory environments, because of the compactness of the associated technology.^3^

Human operators are a vital component of systems to maintain their performance at an appropriate level. Moreover, they have been commonly found in controlling workplaces for many years.^14^ van Daalen et al^15^ indicated that mismatch between MW and capabilities of worker can cause work-related stress. If a human operator experiences extensive levels of MW in daily work conditions without enough rest time, health problems such as chronic stress, depression, or burnout will, therefore, be happen.^4^ When the task is more demanding and complex, the operators should work more to accomplish it.^16^ It is also important to evaluate when and why the operator’s MW increased during system operation.^17^ Usually, operators managing complex work environments may be exposed to different stressors, representing quick dangers to safety, performance and well-being as well as posing long-term consequences for their health.^18^ In addition, it is important to evaluate MW in a real work condition to prevent probable mental disorders and maintain mental health, but most of researches have been carried out to discriminate different levels of MW in a laboratory condition.^4^ The operators of control centers have a basic activity such as monitoring the functioning of the substation, where it is essential to make decisions and process information continuously.^19^ A city traffic control center, cement control center and power plant control center are workplaces in which it is important to give description about their operators’ MW levels and there is not much study in this area. The ECG and electroencephalogram (EEG) signal recording may be collected even during real work tasks inside subjective measures at least in mentioned tasks. This information can be used to evaluate MW levels that operators experience and in unacceptable condition probable risk of mental disorders and human error decreases by optimizing work demands.^20^ In this study, we aimed to apply approaches such as NASA-TLX and physiological measures (e.g., ECG and EEG) to evaluate operator’s MW during a real working condition at a city traffic control center, cement control center and power plant control center in understanding and quantifying MW level.

## Materials and Methods

### Participants

This study was a cross-sectional research conducted from June 2014 to February 2015 at the cement, city traffic control and power plant control centers. First, we wanted to conduct an experiment among all control centers operators, but some of them did not cooperate. We explained the aim of the study and, thereafter, 40 healthy male operators agreed to participate in the study. The age and work experience of them were 32.63 (SD: 0.57) and 5.70 (SD: 0.44) years, respectively. The number of operators in power plant, city traffic and cement control centers were 16 (with mean age of 34.64 [SD: 0.51] and work experience of 6.70 [SD: 0.33] years), 16 (with mean age of 29.40 [SD: 2.61] and work experience of 3.30 [SD 0.80] years) and eight (with mean age of 33.80 [SD: 0.60] and work experience of 7.10 [SD: 0.27] years) respectively. They were paid for their participation in the experiment. All operators were right handed with normal or corrected-to-normal vision and hearing and had no diseases. They read and signed the consent form before the experiment. The experiment was designed to investigate MW in resting, low mental workload (LMW), high mental workload (HMW) and recovery conditions.

### Procedure

**Figure 1:**
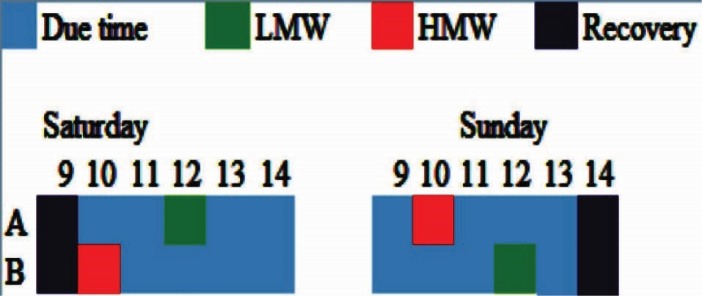
An example of the experimental procedure.

To evaluate MW, we selected operators of the cement, city traffic, and power plant control centers. All operators almost permanently monitored and controlled their system functions or equipments such as temperature, pressure, fuel consumption, ampere changes, traffic density and so on from morning to the end of their shift, and if an accident or special event occurs, they try to reestablish the normal condition of their system. Each operator uses monitors on his desk and monitors continuously vital parameters of the system during his work shift. We interviewed the operators and supervisors in order to characterize their real working conditions. It was recognized that they experienced two different conditions: first, situations in which the mental work is low and, second, situations in which the mental work is high. Based on their statements, LMW and HMW occur during each shift work. Based on this information, measurements related to systems condition were conducted between 9:00 am and 14:00 pm. For example (Figure 1), on Saturday for operator A, ECG and EEG signals were recorded in rest and LMW blocks and for operator B, ECG and EEG signals were recorded in rest and HMW blocks. The next day (Sunday) for operator A, ECG and EEG signals were recorded in HMW and recovery blocks and for operator B, ECG and EEG signals were recorded in LMW and recovery blocks. It is necessary to mention that the NASA-TLX questionnaire was completed immediately by each operator after LMW (or HMW) block. Also, there were no accident and no severe work condition change that evoke rapid MW change during the experiment. The experimenter specified the day with LMW and HMW conditions for each operator, in which the following steps were performed, respectively:

1. Before implementation of the experiment, each operator was provided with the necessary information and descriptions, mainly about the ECG, EEG measurements as well as about how to complete the NASA-TLX questionnaire. Operators’ dominant working postures were a sedentary one in that they had to continuously monitor system status. In order to prevent the system from committing errors and failures during measurement procedure, the experimenter asked the operators to have the least possible amount of movements and avoid talking to his colleagues while physiological indices were being recorded.
2. ECG and EEG electrodes were attached.
3. For each operator, ECG and EEG signals were recorded for 5 minutes in rest condition with open eyes in a quiet room. For 5 minutes, the operator’s colleagues carried out the task on behalf of him.
4. For each operator, during monitoring of LMW (or HMW) condition, ECG and EEG signals were recorded for 5 minutes. After that, the experimenter asked each operator to complete the NASA-TLX questionnaire.
5. For each operator, ECG and EEG signals were recorded for 5 minutes at the end of the shift with open eyes in a quiet room.

### Subjective ratings

The NASA-TLX was used to evaluate subjective workload of the operators. This tool was recommended after three years of extensive research on the physical and mental tasks in different jobs in over 40 simulation laboratories. This index is a multidimensional method with various evaluation degrees, which provides a self-evaluation model to estimate MW through use of six subscales including (MD, mental demand; PD, physical demand; TD, temporal demand; OP, own performance; EF, effort; and FR, frustration). Each subscale has been presented as an 12-cm line with a title (e.g., Mental Effort) and bipolar descriptors at each end (e.g., High/Low). Numerical values have not been displayed, but values ranging from 1 to 100 have been assigned to scale positions during data analysis.^5^ The process of MW evaluation through NASA-TLX involves three steps. At the first step, weight in each of the six subscales is determined to reveal the priority of the six subscales of TLX. At this step, all subscales are self-evaluated and selected by the operator in a paired form and in 15 different comparisons, and then, each workload dimension is scored 0-5. At the second step, to allocate the rating of workload, each of the six subscales is rated with the goal of determination of each scale’s effect on the MW. At this step, the operator scores each of these six subscales from 1 to 100 based on his/her own working condition. At the third and the last step, after determination of weight and rating in previous steps, total MW is calculated in the range 1-100 through the following formula: “Weighted Workload (WWL) is Ʃ(rating × weight)/15.”^21^

The validity and reliability of this tool have been previously confirmed.^5^ In order to determine the face validity of the NASA-TLX a back-ward translation method have been used.^22^ Also, in two studies conducted for the evaluation of nurses’ MW in the intensive care unit in Tehran and Isfahan, its reliability was confirmed (Cronbach alpha coefficient=0.847 and 0.83 respectively).^22,23^

### Physiological measurement

**Figure 2:**
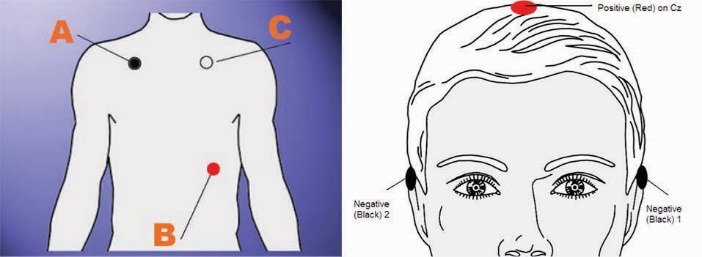
Electrode positions for ECG (left) and EEG (right). Reprinted with permission from Mind Media BV, The Netherlands.

A NeXus-4 of Mind Media BV was used for data collection. This system allowed acquisition of signals, including EEG, ECG, EMG, EOG, etc. The acquired signals were wirelessly transmitted, using Bluetooth wireless communication, for online monitoring and data storage. Online graphic presentations of the physiological parameters and retrieval of database, data processing, digital filtering, report of trends and statistical analysis functions were provided by a compatible software (BioTrace+^®^, Mind Media BV, Roermond-Herten, The Netherlands). Two physiological parameters from ECG, and EEG were recorded for this study. Channels operating at two sample frequencies of 1024 and 256 Hz were used to measure heart rate (ECG) and brain activity (EEG), respectively. For measuring these electrophysiological signals, NeXus uses carbon coated cables with active shielding. Effectively that means very clean signals with virtually no movement artifacts. NeXus uses active noise cancellation technology to lessen movement artifact and external interference and it also provides very good ECG and EEG signals. The environmental noise is electronically subtracted from the EXG signal, resulting in very clean signals with very few artifacts. Movement artifact is virtually absent; 50/60 Hz noise is very low. Biotrace+ uses mostly the ECG signal from the NeXus to measure HR and HRV. The ECG was recorded using three Ag–AgCl electrodes. The electrodes were placed at the distal part of sternum and at the sixth rib in the left axilla (Figure 2 left). HRV, which refers to the beat-to-beat alterations in HR, was evaluated on the basis of ECG recordings during all the four conditions. The following features were calculated from recorded ECG signals: the mean value of the HR (Mean HR), the standard deviation of the RR intervals (SDNN), the root mean square of successive difference of the RR intervals (RMSSD), and the ratio of the Low Frequency over the High Frequency (LF/HF). For EEG electrode placement, we used the international 10-20 EEG system. We placed the EEG electrodes on the head using NuPrep (for skin preparation) and 10-20 EEG paste. We used one channel of EEG. For a basic one channel EEG signal recording typically the left ear (or mastoid) was used for the reference electrode. An electrode was placed on Cz and the ground electrode was placed on the right ear (Figure 2 right).

### Data analysis

The difference between subjective responses at LMW and HMW conditions for all subscales and overall workload (WWL) of the NASA-TLX was analyzed using two-way analysis of variance (ANOVA). All physiological parameters were analyzed applying repeated measures ANOVA to examine the differences between task and interactions with step of measuring conditions (resting, LMW, HMW and recovery). The Greenhouse–Geisser correction was applied. The factor of the task included the factor between operators. The effect size (partial etta sqaured η2) was reported and Bonferroni multiple comparison method was used when main effects were significant. The statistical analysis was conducted using SPSS version 21.0. A 5% significance level was adopted in all tests.

## Results

### Subjective responses

**Figure 3:**
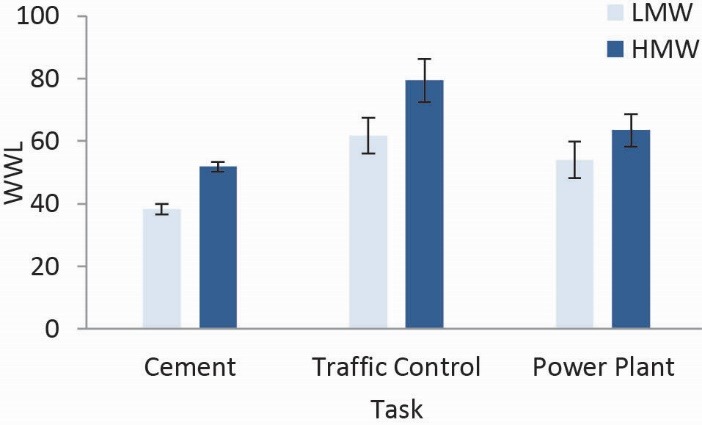
Changes in NASA-TLX score across two conditions among tasks. Error bars indicate standard errors of the mean.

**Figure 4:**
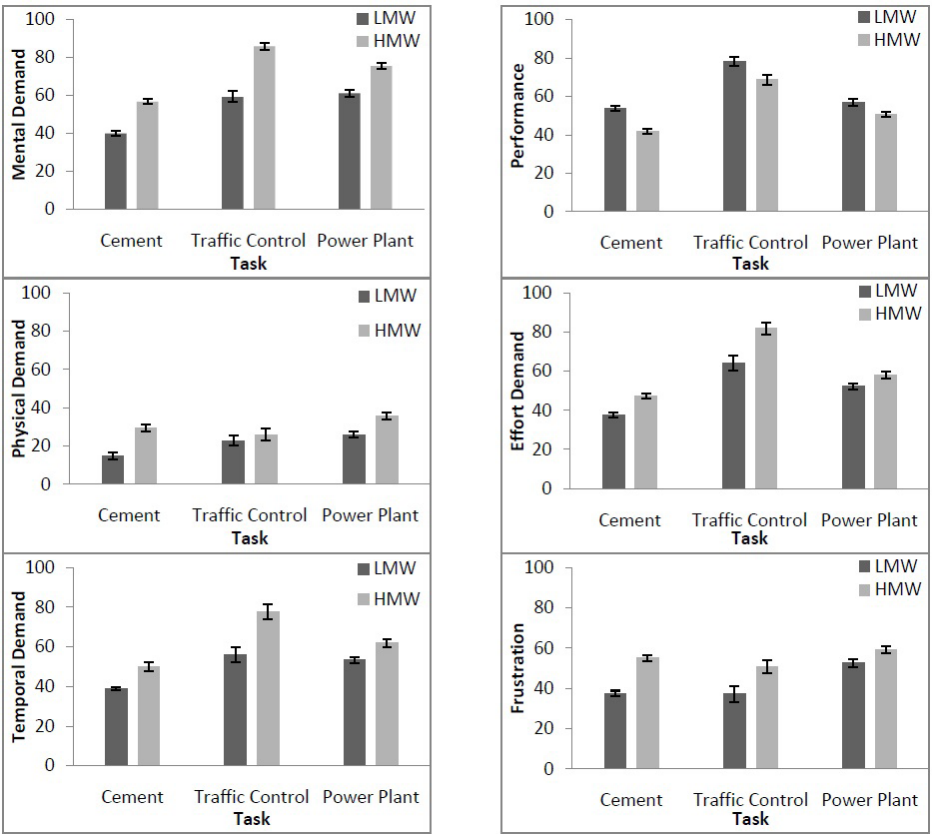
Changes in NASA-TLX subscales across two conditions among tasks. Error bars indicate standard errors of the mean

The results of the subjective ratings of workload measured by NASA–TLX across LMW and HMW conditions for all operators have been summarized in Table 1. Weighted workload (WWL) in LMW and HMW conditions were 52.1 and 63.4, respectively. Based on the operators’ subjective responses, in LMW condition OP and in HMW condition MD had dominant importance and in LMW and HMW conditions PD had the lowest importance. The results of two way ANOVA indicated significant differences for means of the NASA-TLX and its dimensions between LMW and HMW conditions, except for OP (Table 1). Based on the operators’ subjective responses, WWL score was significantly higher in traffic control task than in other tasks (Figure 3). Changes in NASA-TLX subscales across LMW and HMW conditions among tasks have been illustrated in Figure 4. From the operators’ perspective, the degree of difficulty varied based on each task for LMW and HMW conditions. The results showed that, based on operators’ point of view, MD, TD, OP, and EF subscales in traffic control task and PD, FR subscales in power plant task had dominant importance and MD, PD, TD, OP, and EF in cement control center task and FR in traffic control task had the lowest importance. Also, in two conditions, based on operators’ point of view, overall means for PD had the lowest score for all tasks. In the traffic control task for LMW condition OP, EF, and MD and in the HMW condition MD, EF and TD had dominant importance, respectively. A repeated measures ANOVA revealed that increasing MW had a significant effect on the operators’ subjective responses in LMW compared to HMWF ([1,53]=216.303, *P*<0.001, ε η2=0.803). Task-MW interaction effect on operators’ subjective responses was significant (F [3, 53]=12.628, *P*<0.001, η2=0.417). A bonferroni post hoc test showed that there was a significant differences for operators’ subjective responses in all tasks (*P*<0.001).

**Table 1:** Comparison of subjective variables Mean ± SE across LMW and HMW conditions

**NASA TLX**	**LMW**	**HMW**	**ANOVA result**	
			**MW** ***P *** **value**	**MW effect size**
MD	54.4 ± 1.43	73.3 ± 1.52	0.001	0.739
PD	21.4 ± 1.08	30.3 ± 1.22	0.016	0.519
TD	49.4 ± 1.41	63.5 ± 1.81	0.001	0.691
OP	60.0 ± 1.25	56.7 ± 2.00	0.172	0.092
EF	51.3 ± 1.75	63.1 ± 1.94	0.03	0.481
FR	44.9 ± 1.53	55.8 ± 1.16	0.008	0.559
WWL	52.1 ± 1.20	63.4 ± 1.49	0.012	0.517

Abbreviations‏: MD, mental demand; PD, physical demand; TD, temporal demand; OP, own performance; EF, effort; FR, frustration; WWL, weighted workload; MW, mental workload; ANOVA, analysis of variance; SE, standard error.

### Physiological measures

**Figure 5:**
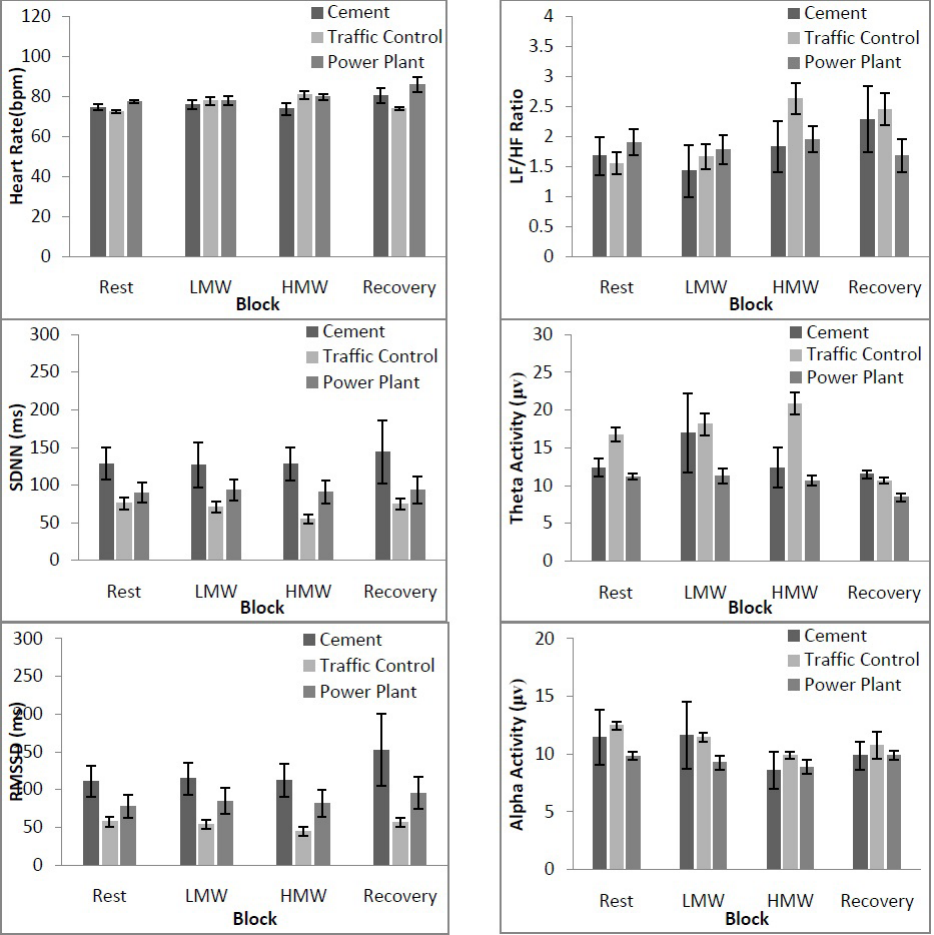
Changes in physiological indices across four blocks among tasks. Error bars indicate standard errors of the mean.

The mean values including standard errors of all operators’ physiological indices in four blocks have been illustrated in Table 2. The results of repeated measures ANOVA indicated that increasing MW has a significant main effect on; HR, LF/HF ratio, theta and alpha band activity. Changes in physiological indices across four blocks between tasks have been demonstrated in Figure 5. A repeated measures ANOVA revealed that increasing MW has a significant effect on heart rate and there is significant differences between heart rate during conducting mental task compared to rest and recovery condition F([1.864,98.812]=4.643, *P*=0.014, η2=0.608). Task-MW interaction effect on heart rate was significant (F [5.593, 98.812]=3.063, *P*=0.010, η2=0.648). A Bonferroni post hoc test showed that there was a significant differences for operators HR in cement and traffic control tasks (*P*=0.006), traffic control and power plant tasks (*P*<0.001). A repeated measures ANOVA showed that increasing MW has not a significant effect on SDNN feature; (F [1.152, 61.032]=1.385, *P*=0.248, η2=0.025). Task-MW interaction effect on SDNN was not significant (F [3.455, 61.032]=0.547, *P*=0.676, η2=0.030). Results of repeated measures ANOVA indicated that increasing MW has not a significant effect on RMSSD feature; (F [1.105, 58.586]=3.124, *P*=0.079, η2=0.056). Task-MW interaction effect on RMSSD was not significant (F [3.316, 58.586]=0.775, *P*=0.524, η2=0.042). The analysis of repeated measures ANOVA showed that increasing MW had a significant effect on LF/HF ratio; (F [1.700, 90.121]=3.404, *P*=0.045, η2=0.160). Task-MW interaction effect on LF/HF ratio was not significant (F [5.101, 90.121]=2.216, *P*=0.058, η2=0.11). A repeated measures ANOVA revealed that increasing MW had a significant effect on theta band activity and there were significant differences in theta band activities during conducting mental task compared to rest and recovery conditions (F (2.407,127.594)=12.843, *P*<0.001, η2=0.195). Task-MW interaction effect on theta band activity was significant (F [7.222, 127.594]=9.973, *P*<0.001, η2=0.361). The Bonferroni post hoc test showed that there was a significant difference for operators’ theta band activity in cement and traffic control (*P*=0.006), traffic control and power plant (*P*<0.001). A repeated measures ANOVA revealed that increasing MW had a significant effect on alpha band activity and there were significant differences between alpha band activity during conducting mental task compared to rest and recovery conditions (F(1.737, 92.064)=49.709, *P*<0.001, η2=0.484). Task-MW interaction effect on alpha band activity was not significant (F [5.211, 92.064]=0.736, *P*=0.604, η2=0.040).

**Table 2:** Comparison of physiological variables Means ± SE across resting, LMW, HMW, and recovery conditions

**Variables**	**R**	**LMW**	**HMW**	**REC**
HR (Beats/min)	75.9 ± 0.62	77.8 ± 1.04	79.7± 1.00	80.0 ± 1.41
SDNN (ms)	91.8 ± 7.78	94.6 ± 9.38	87.3 ± 8.73	109.0 ± 11.00
RMSSD (ms)	77.5 ± 8.17	83.2 ± 9.83	76.8 ± 9.41	108.7 ± 13.3
LF/HF ratio	1.7 ± 0.11	1.8 ± 0.14	2.1 ± 0.13	2.1 ± 0.15
Theta (μv)	13.1 ± 0.63	14.1 ± 0.99	15.5 ± 1.10	10.4 ± 0.31
Alpha (μv)	10.8 ± 0.39	10.4 ± 0.49	9.2 ± 0.33	10.28 ± 0.40

## Discussion

This study was conducted to evaluate the MW of the operators in a cement, traffic control and power plant control centers while they were monitoring vital parameters of their systems. The present study investigated the cardiovascular indices, brain activity, and subjective responses of the operators to their MW.

Using NASA-TLX, all operators stated that the task demands of the HMW condition were higher than those of the LMW condition. In the LMW condition, OP was the highest, and in the HMW condition, MD was the highest. This means that the operators tried to maintain their performance at the highest level in the LMW condition, and when task demands increased, MD increased as well. The high the average mental requirement, the more the operator felt that his task was demanding. The findings showed that cement control center operators experience low to moderate MW in two conditions and in this situation probable of mental disorders will be decreased. Furthermore, in the LMW condition power plant operators experienced high MD compared to traffic control and cement operators. In the HMW condition, the traffic control operators experienced high MD compared to the power plant and cement operators. It seems that when MW increased, the operators working at traffic control center experienced more mental stress, which could lead to mental disorders under these circumstances. It was observed that the demand for physical effort was indeed very small for all control center operators. Because of good OP in the LMW condition, the operators had low levels of FR, and in this way they may have experienced lower mental stress. However, during the HMW condition, especially in the traffic control and power plant control centers, MD and the level of FR increased with an increase in task demands, which may have caused operators to experience more stress while performing their tasks.

In this study, MW had a marked impact on the physiological parameters with increasing task demands during work sessions, namely during LMW and HMW conditions compared with resting and recovery conditions. Moreover, the shift from LMW to HMW was clearly reflected in HR, LF/HF ratio, theta and alpha bands activity. A statistically significant difference was observed for HR, LF/HF ratio, and theta and alpha bands activity. Cinaz et al^4^ indicated that the LF/HF ratio significantly increases with increased MW. In the study of Reimer and Mehler,^24^ the pattern of change in HR with increased MW was highly consistent between field and simulator conditions. Hwang et al^25^ stated that HR and LF/HF ratio increased with increasing task complexity. Knaepen et al^26^ indicated that HR significantly increased with increasing MW during walking. Besides, Zhang et al^27^ stated that mental arithmetic tasks were found to significantly increase HR. Our results were consistent with those of the above mentioned studies.

In this study, it was observed that by increasing task demands and more visual information processing EEG theta band activity increased. Similarly, during a flight scenario investigated by Hankins and Wilson,^28^ the theta band of the EEG increased in mental calculation conditions. In addition, Fairclough et al^29^ reported that theta activity increased in response to increased task demands.

The results from the EEG showed that the EEG alpha band amplitude was lower during the HMW condition than the LMW condition. However, these differences were statistically significant. The graph showed that the mean amplitude of the EEG alpha band tended to decrease as the task demands increased. The decreased EEG alpha band amplitude showed that the operators of the traffic control center and power plant experienced more fatigue. This finding confirmed the results of prior study in which inverse relationship between alpha band activity and task difficulty was observed.^30^ Also, in the study by Ryu and Myung^3^ the alpha band suppression indicated a systematic decrease, as the difficulty of the arithmetic task increased. Thus, this study indicated that operators of traffic control and power plant control centers experienced more mental stress as mental demand of tasks increased. This mental stress probably will occur among the operators because of changes in heart rate, LF/HF ratio, EEG theta and alpha bands in every day of working hours.

### Limitations

Some limitations of this study should be mentioned. All operators in the control centers were men. Thus, the study did not address the effects of sex, nor did it report any sex differences while quantifying the effects of MW on physiological and subjective responses. Future research should try to evaluate the MW of operators in workplaces with both men and women considering their shift work patterns. We recorded physiological indices for 5 minutes in LMW and HMW conditions, so measuring physiological indices in workplaces, in which operators experience different MW levels for eight hours during shift work, may contribute to improve the design of ergonomic interventions, such as task and workstation design, in order to reduce mental stress.

## Conclusion

This study was conducted in three control centers during real working conditions. Our experiment showed that working in HMW condition led to an increase in both subjective and some physiological responses such as HR, LF/HF ratio, and theta and alpha band. In addition, OP was the highest and most consistent sub-scale when working with LMW, while it was diminished with HMW. MD subscale as measured by NASA-TLX, was the highest and most consistent when working with HMW condition for the power plant and traffic control tasks. Increasing mental demand had a significant effect on physiological variables. The mean age and work experience of the operators were 32.63 and 5.7 years, respectively. It is expected that with increasing work experience mental fatigue and stress will increase and the mental health of the operators of the traffic control and power plant will probably deteriorate. Because all operators experience both HMW and LMW conditions during their shifts, it might be necessary to maintain OP and MD at acceptable levels to manage the mental health of operators. The traffic control center and power plant directors are proposed to apply an administrative control for managing mental health among operators in the future. For example, we suggested decreasing the time spend to monitor parameters of system by each operator in HMW condition. The findings reported in this study may be generalizable to complex work systems operators but some of the results are likely to be due to the characteristics of the evaluated control centers. Finally, through analysing MW, the control center director can organize the human resources for each MW condition to sustain the appropriate performance as well as improve system functions.

## Ethical approval

The study protocol was approved by the Ethics Committee of Hamadan University of Medical Sciences.

## Competing interests

The authors declare that there is no conflict of interest.

## Author contributions

MF performed data collection, interpreted the results, and prepared this manuscript. MM took the first initiative on the outline and designed overall this study. RH and SM contributed to critical revision of important intellectual content. ARS analyzed the results. All authors read and approved the final manuscript.

## Acknowledgments

Thanks to Deputy of Research and Technology of Hamadan University of Medical Sciences for approving and funding this research work. Also, we would like to thank the operators of the control centers for their time and engagement with this study.

## References

[1] Kum S, Furusho M, Duru O, Satir T (2007). Mental workload of the VTS operators by utilising heart rate. TransNav.

[2] Jou YT, Yenn TC, Lin CJ, Yang CW, Chiang CC (2009). Evaluation of operators’ mental workload of human–system interface automation in the advanced nuclear power plants. Nuclear Engineering and Design.

[3] Ryu K, Myung R (2005). Evaluation of mental workload with a combined measure based on physiological indices during a dual task of tracking and mental arithmetic. Int J Ind Ergon.

[4] Cinaz B, ArnrichB ArnrichB, La Marca R, Tröster G (2013). Monitoring of mental workload levels during an everyday life office-work scenario. Pers Ubiquitous Comput.

[5] Rubio S, Díaz E, Martín J, Puente JM (2004). Evaluation of subjective mental workload: A comparison of SWAT, NASA‐TLX, and workload profile methods. Appl Psychol.

[6] de Winter JC (2014). Controversy in human factors constructs and the explosive use of the NASA-TLX: a measurement perspective. Cogn Tech Work.

[7] Hart SG, Staveland LE (1988). Development of NASA-TLX (Task Load Index): Results of empirical and theoretical research. Adv Psychol.

[8] Tran TQ, Boring RL, Dudenhoeffer DD, Hallbert BP, Keller MD, Anderson TM, editors. Advantages and disadvantages of physiological assessment for next generation control center design. Human Factors and Power Plants and HPRCT 13th Annual Meeting; 2007.

[9] Jorna P (1993). Heart rate and workload variations in actual and simulated flight. Ergonomics.

[10] Roscoe AH (1993). Heart rate as a psychophysiological measure for in-flight workload assessment. Ergonomics.

[11] Reyes del Paso GA, Langewitz W, Mulder LJ, van Roon A, Duschek S (2013). The utility of low frequency heart rate variability as an index of sympathetic cardiac tone: a review with emphasis on a reanalysis of previous studies. Psychophysiology.

[12] Käthner I, Wriessnegger SC, Müller-Putz GR, Kübler A, Halder S (2014). Effects of mental workload and fatigue on the P300, alpha and theta band power during operation of an ERP (P300) brain–computer interface. Biol Psychol.

[13] Lean Y, Shan F (2012). Brief review on physiological and biochemical evaluations of human mental workload. Human Factors and Ergonomics in Manufacturing & Service Industries.

[14] Balfe N, Sharples S, Wilson JR (2015). mpact of automation: measurement of performance, workload and behaviour in a complex control environment. Appl Ergon.

[15] van Daalen G, Willemsen TM, Sanders K, van Veldhoven MJ (2009). Emotional exhaustion and mental health problems among employees doing “people work”: The impact of job demands, job resources and family-to-work conflict. Int Arch Occup Environ Health.

[16] Vidulich MA, Tsang PS (2015). The confluence of situation awareness and mental workload for adaptable human–machine systems. J Cogn Eng Decis Mak.

[17] Jo S, Myung R, Yoon D (2012). Quantitative prediction of mental workload with the ACT-R cognitive architecture. Int J Ind Ergon.

[18] Sauer J, Nickel P, Wastell D (2013). Designing automation for complex work environments under different levels of stress. Appl Ergon.

[19] Vitório DM, Masculo FS, Melo MO (2012). Analysis of mental workload of electrical power plant operators of control and operation centers. Work.

[20] Fallahi M, Motamedzade M, Heidarimoghadam R, Soltanian AR, Miyake S (2016). Effects of mental workload on physiological and subjective responses during traffic density monitoring: a field study. Appl Ergon.

[21] Habibi E, Taheri MR, Hasanzadeh A (2015). Relationship between mental workload and musculoskeletal disorders among Alzahra Hospital nurses. Iran J Nurs Midwifery Res.

[22] Mohammadi M, Mazloumi A, Kazemi Z, Zeraati H (2015). Evaluation of Mental Workload among ICU Ward’s Nurses. Health Promot Perspect.

[23] Safari S, Mohammadi Bolban Abad H, Kazemi M (2013). Evaluation mental work load in nursing critical care unit with NASA-TLX index. Health Syst Res.

[24] Reimer B, Mehler B (2011). The impact of cognitive workload on physiological arousal in young adult drivers: a field study and simulation validation. Ergonomics.

[25] Hwang SL, Yau YJ, Lin YT, Chen JH, Huang TH, Yenn TC (2008). Predicting work performance in nuclear power plants. Saf Sci.

[26] Knaepen K, Marusic U, Crea S, Guerrero CDR, Vitiello N, Pattyn N (2015). Psychophysiological response to cognitive workload during symmetrical, asymmetrical and dual-task walking. Hum Mov Sci.

[27] Zhang J, Yu X, Xie D (2010). Effects of mental tasks on the cardiorespiratory synchronization. Respir Physiol Neurobiol.

[28] Hankins TC, Wilson GF (1998). A comparison of heart rate, eye activity, EEG and subjective measures of pilot mental workload during flight. Aviat Space Environ Med.

[29] Fairclough SH, Venables L, Tattersall A (2005). The influence of task demand and learning on the psychophysiological response. Int J Psychophysiol.

[30] Gevins A, Smith ME, Leong H, McEvoy L, Whitfield S, Du R (1998). Monitoring working memory load during computer-based tasks with EEG pattern recognition methods. Hum Factors.

